# Increased serum nesfatin-1 levels in patients with acromegaly

**DOI:** 10.1097/MD.0000000000022432

**Published:** 2020-10-02

**Authors:** Yakun Yang, Song Han, Zuocheng Yang, Pengfei Wang, Chang-Xiang Yan, Ning Liu

**Affiliations:** Department of Neurosurgery, Sanbo Brain Hospital, Capital Medical University, China.

**Keywords:** acromegaly, disordered metabolism, growth hormone-secreting pituitary adenoma, Nesfatin-1/nucleobindin2

## Abstract

Nesfatin-1 was identified as a satiety factor involved in the regulation of metabolism. Altered levels of circulating nesfatin-1 had been observed in a variety of diseases characterized by energy imbalance. However, there was no published data about nesfatin-1 levels in acromegaly.

We evaluated serum nesfatin-1 levels in 13 patients with acromegaly at baseline and postoperatively, and in 21 age- and body mass index (BMI)-matched healthy subjects.

Compared with the healthy subjects, patients with acromegaly had significantly increased levels of serum insulin, high-density lipoprotein cholesterol, triglyceride, and growth hormone (GH). Moreover, the acromegaly group had nesfatin-1 levels higher than controls (1.96 ± 0.56 ng/mL vs 0.61 ± 0.10 ng/mL, *P* = .004). There was a positive correlation of serum nesfatin-1 levels with diastolic blood pressure (*r* = 0.579, *P* = .038) and homeostasis model assessment of insulin resistance (HOMA-IR) (*r* = 0.598, *P* = .031) in patients with acromegaly. While a successful surgery decreased serum GH levels, the serum nesfatin-1 levels did not change in acromegaly (*P* = .965). At last, we compared serum GH/nesfatin-1 levels with predictive markers for aggressive behaviors in pituitary adenomas. There was no relationship between serum nesfatin-1 levels and tumor's size, Ki-67 index, mutant p53, or MGMT proteins. However, increased serum GH levels were positively correlated with tumors’ size (*P* = .023) and mutant p53 proteins expression (*P* = .028).

Circulating nesfatin-1 was increased in acromegaly, which was involved in metabolism regulation.

## 1 Introduction

Nesfatin-1 has been identified as an anorectic peptide, which processed from nucleobindin2 (NUCB2) in the hypothalamus.^[1]^ The origin of circulating nesfatin-1 was mainly from gastrointestinal tract and adipose tissue, where involved in pathophysiological process,^[2]^ participating in the regulation of metabolism and the improvement of insulin sensitivity.[^3^,^4^,^5^] Plasma nesfatin-1 are decreased in patients with type 2 diabetes.^[6]^ Later, altered levels of circulating nesfatin-1 had been observed in a variety of disease characterized by disordered metabolism, which are type 2 diabetes,[^6^,^7^] gestational diabetes,^[8]^ polycystic ovary syndrome,^[9]^ and hyperthyroidism.[^10^,^11^] However, these results suggested that circulating nesfatin-1 levels were inclined to change due to the disordered metabolism, which was characterized by impaired fasting glucose, insulin resistance, and abnormal lipid metabolism.

Acromegaly is a relatively uncommon disease, with an estimated prevalence at 1:140,000–250,000.^[12]^ The excess of growth hormone (GH) production could lead to insulin resistance, impaired glucose tolerance, and even diabetes.^[12]^ Ghrelin, another appetite regulatory peptide also had changed levels in acromegaly.[^13^,^14^] The direct relation between growth hormone and the nesfatin-1 has not been studied yet. The growth hormone and the nesfatin-1were both related to insulin resistance and Ghrelin, suggests the potential connections of growth hormone and the nesfatin-1. So, the circulating nesfatin-1 levels were likely to change in acromegaly, as it participated in the regulation of metabolism.

Moreover, recent studies suggested a crucial role of nesfatin-1 in the tumor development.^[15]^ We hypothesized that altered levels of nesfatin-1 caused by disordered metabolism in acromegaly, may further promote the tumor development. In the present study, we first detected the serum nesfatin-1 levels in acromegaly, and correlated the hormone levels with tumors’ clinicopathological factors.

## 2 Patients and methods

### 2.1 Patients and samples

Group 1 (healthy subjects) fasting serum nesfatin-1 level was detected in 21 healthy subjects (females/males, 10/11; age, 39.81 ± 2.45 years). The mean body mass index (BMI) was 24.33 ± 0.39 kg/m^2^.

Group 2 (acromegaly) 13 patients with acromegaly (female/male 9/4; age 41.23 ± 3.61 years) were involved in the study. The diagnosis of acromegaly was based on the clinical presentation, magnetic resonance imaging (MRI) image and the serum levels of GH. While 10 patients were newly diagnosed, 3 patients experienced tumor recurrence after at least one operation. Additionally, 2 patients showed the invasion of cavernous sinus by the tumor with MRI. These 5 patients’ tumors were classified as aggressive and invasive pituitary adenomas.^[16]^ None of the patients suffered from other endocrinological disease or received any related medications. Among the 13 patients, 11 patients had a surgical treatment. There are 9 cases treated with a transsphenoidal surgery and 2 cases with a craniotomy. All the surgical procedures were successful and no complication occurred.

All the blood specimens for hormone or biochemical analysis were drawn from a forearm vein in the morning after overnight fasting. Postoperational blood samples were collected on the third day postoperatively. Tumor samples used in the study were obtained fresh from operation at Sanbo Brain Hospital. Informed consent was obtained from all patients prior to the study. All experiments using human tissues are approved by the Institutional Review Board of Sanbo Brain Hospital.

### 2.2 Assay

Blood samples were collected after an overnight fast, separated immediately by centrifugation at 1000 × *g* for 10 minutes at 4 °C, and stored at −80 °C until assay. Serum nesfatin-1 levels were investigated using Enzyme Linked Immunosorbant Assay (Phoenix Pharmaceuticals, Belmont, CA). The linear range of the assay was 0.78 to 50 μg/L. The detection limit was increased by adding 0.78 ng/mL pure nesfatin-1 to the samples and subtracting this from the total amount measured at the end of the experiment, as we previously did.^[6]^ Intra- and inter-assay CV were <5% and <15%, respectively. Serum GH levels (RIA kit, Siemens DPC2000, 154 Wittelsbacherplatz 280333 München) and insulin levels (Elisa kit, ROCHE E601, 180953 F.Hoffmann-La Roche AG Konzern-Hauptsitz Grenzacherstrasse 124 CH-4070 Basel) were measured using a commercially available RIA and an Elisa kit respectively.

### 2.3 Immunohistochemistry

Immunostaining was performed on 4-μm-thick sections of formalin-fixed and paraffin-embedded samples. The sections were dewaxed by xylene, dehydrated by gradient ethanol, and then rehydrated with PBS (Phosphate Buffered Saline). To facilitate antigen retrieval, the sections were incubated in 10 mM of citric acid (pH 6.0) at 120 °C for 150 seconds. We used 3% hydrogen peroxide to neutralize endogenous peroxidases of the samples for 15 minutes. Primary antibodies against p53 (1:100 Invitrogen), nuclcar- associated antigen Ki-67 (1:200 Invitrogen), and O^6^-methylguanine DNA methyltransferase (MGMT) (1:150 Invitrogen) were then applied overnight at 4 °C. The cutoff values were 3% for nuclcar- associated antigen Ki- 67, 5% for p53, and 10% for MGMT respectively.

### 2.4 Measurement

The tumor size was evaluated by the MRI before the operation. BMI was calculated as weight (kg)/height (m)^2^. The homeostasis model assessment of insulin resistance (HOMA-IR) was studied using following equations (HOMA-IR = fasting insulin (mU/L) × fasting glucose [mmol/L]/22.5). The quantitative insulin sensitivity check index (QUICKI) was calculated as followed (QUICKI = 1/[log fasting glucose {mg/dL} + log fasting insulin {mU/L}]).

### 2.5 Statistics

Data were expressed as mean ± SEM. All data analysis was carried out by Statistical Product and Service Solutions 22.0. Comparisons between 2 groups were analyzed using the Mann–Whitney *U* test. Differences between hormonal levels before and after operation were made with the paired *t* test. The correlations between different parameters were performed with the simple regression analysis. The predictive value of serum nesfatin-1 for acromegaly was analyzed by receiver operating characteristic (ROC) curve analysis. *P*< .05 was taken as statistically significant.

## 3 Results

### 3.1 Baseline

**Figure 1:**
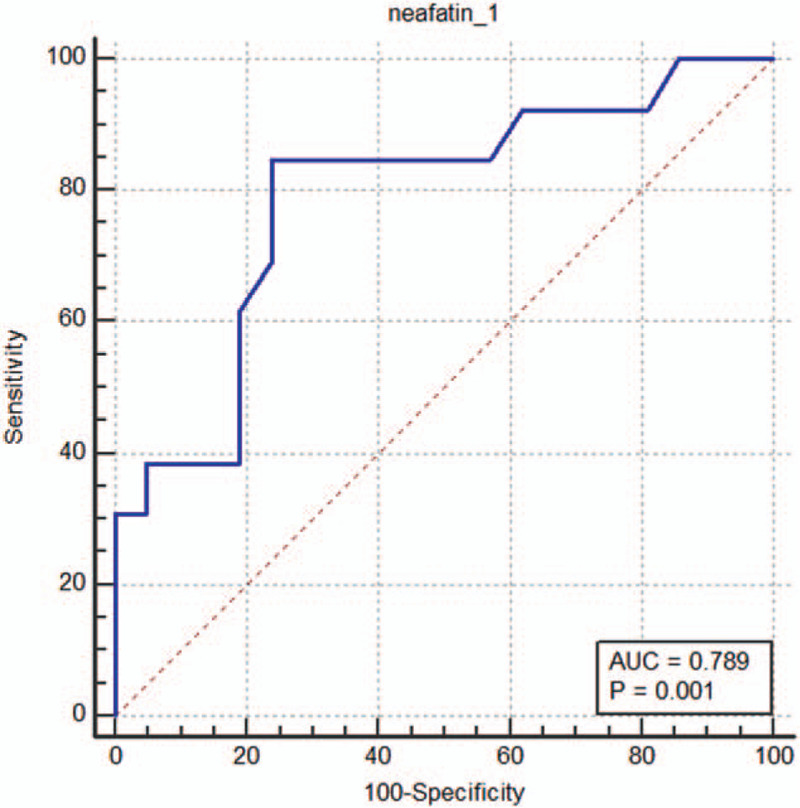
ROC curve for nesfatin-1. The cutoff value of 0.688 ng/mL was determined for the diagnosis of acromegaly. The sensitivity and specificity of the value was 0.846 and 0.762 respectively. ROC = receiver operating characteristic.

The 2 groups were matched for age and sex. BMI in the acromegaly group was higher than that in the normal subjects but not significantly. (1.96 ± 0.56 ng/mL vs 0.61 ± 0.10 ng/mL, *P* = .089) There was no significant difference in systolic pressures and diastolic pressures among the 2 groups. Acromegalic patients had a higher fasting plasma glucose levels compared with the normal subjects (Table 1). However, patients with acromegaly showed significantly elevated serum insulin levels, HOMA-IR and QUICKI values, compared with the other group (Table 1). Acromegalic patients also exhibited significantly an increased level of plasma triglyceride and a decreased level of high-density lipoprotein (Table 1). Finally, we observed an increased level of both serum GH level (37.1 ± 9.44 ng/mL vs 1.12 ± 0.33 ng/mL, *P* = .000) and nesfatin-1 level (1.96 ± 0.56 vs 0.61 ± 0.10 ng/mL, *P* = .004) in the acromegaly group (Table 1). The cutoff value of nesfatin-1 between healthy subjects and acromegalic patients was 0.688 ng/mL. The sensitivity and specificity of the value was 0.846 and 0.762 respectively (Fig. 1).

**Table 1:**
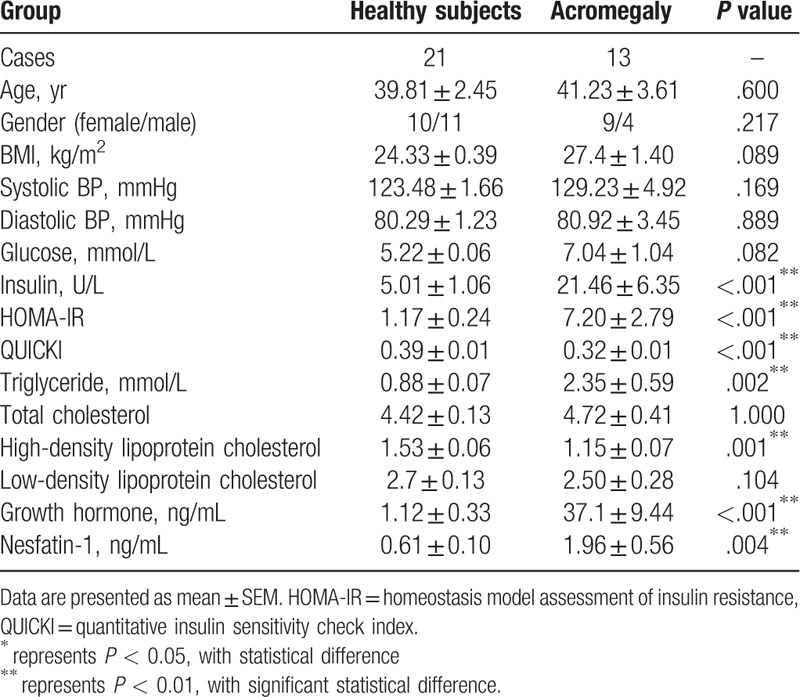
Clinical characteristics and nesfatin-1 levels in healthy subjects and patients with acromegaly.

There is a strong positive correlation between serum nesfatin-1 level and diastolic blood pressure (*r* = 0.579, *P* = .038) or HOMA-IR (*r* = 0.598, *P* = .031) in the acromegaly group (Table 2). However, serum nesfatin-1 level did not correlate with glucose, insulin, total cholesterol, HDL-cholesterol levels or QUICKI values, neither in normal subjects nor with acromegalic patients. No significant correlation was also found between serum nesfatin-1 and GH levels among the 2 groups (Table 2).

**Table 2:**
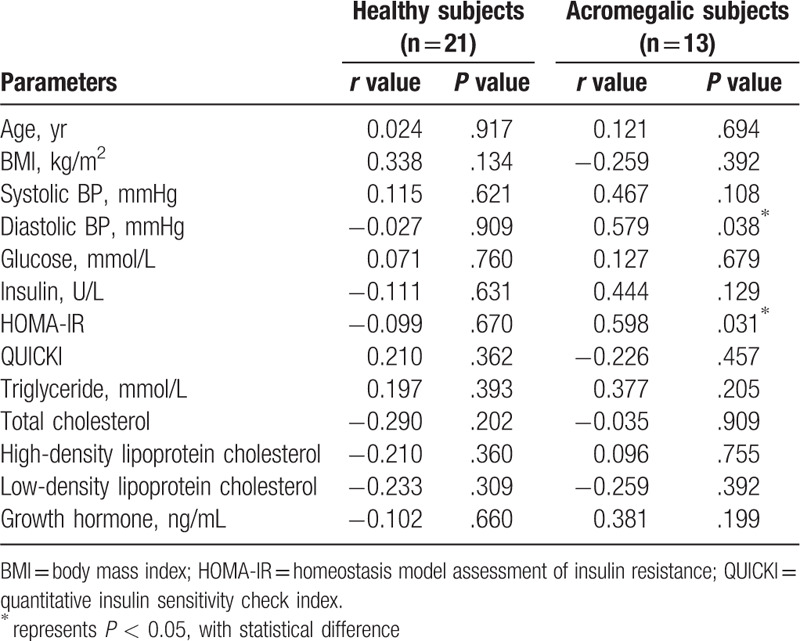
The correlations between Nesfatin-1 and measured parameters in healthy subjects and acromegalic subjects.

### 3.2 Perioperative changes in serum nesfatin-1 levels

**Figure 2:**
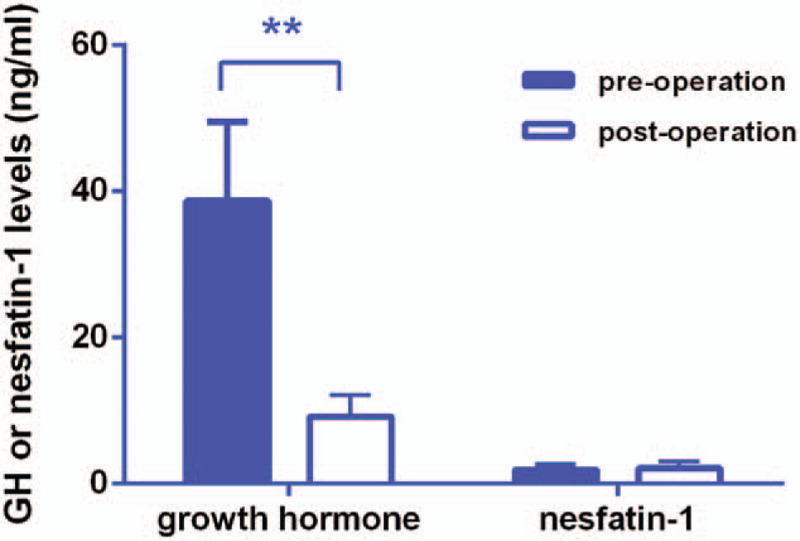
The perioperative serum GH and nesfatin-1 levels in acromegaly. The changes of serum GH and nesfatin-1 levels in the acromegaly group before surgery and on Days 3 after surgery. Data were expressed as mean ± SD, and analyzed by the Mann–Whitney *U* test. Serum GH levels were decreased significantly after the surgery. No significant change of serum nesfatin-1 levels were observed after the surgery. GH = growth hormone.

Serum GH levels in acromegaly decreased significantly after operation. (38.67 ng/mL vs 12.82 ng/mL, *P* = .003, Fig. 2) While there was no change in serum nesfatin-1 levels after operation. (1.91 ng/mL vs 2.14 ng/mL, *P* = .965, Fig. 2). We also observed a significant decrease in serum insulin-like growth factor-1 levels in 6 patients after acromegaly. (906.67 ng/mL vs 706.83 ng/mL, *P* = .033) However, postoperative serum nesfatin-1 levels showed a margin significant correlation with serum GH levels (*r* = 0.600, *P* = .051).

**Figure 3:**
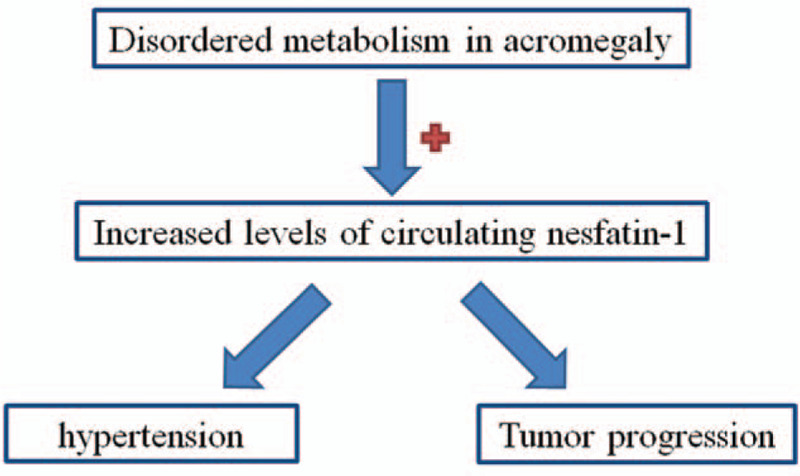
Schematic for the increased levels of nesfatain-1 in acromegaly.

### 3.3 Clinical characteristics of serum nesfatin-1 levels in pituitary adenomas

We further related the serum nesfatin-1 and GH levels to the characteristics of the growth hormone-secreting pituitary adenoma.^[16]^ Serum nesfatin-1 or GH levels did not correlate with tumor's invasiveness or aggressiveness. There was no significant correlation between the serum nesfatin-1 levels and tumor's diameter or the immunostaining of nuclcar- associated antigen Ki- 67, p53, and MGMT (Table 3). However, we observed that serum GH levels coincided with tumor's diameter and p53 mutation whereas no correlation was established for others (Table 3).

**Table 3:**
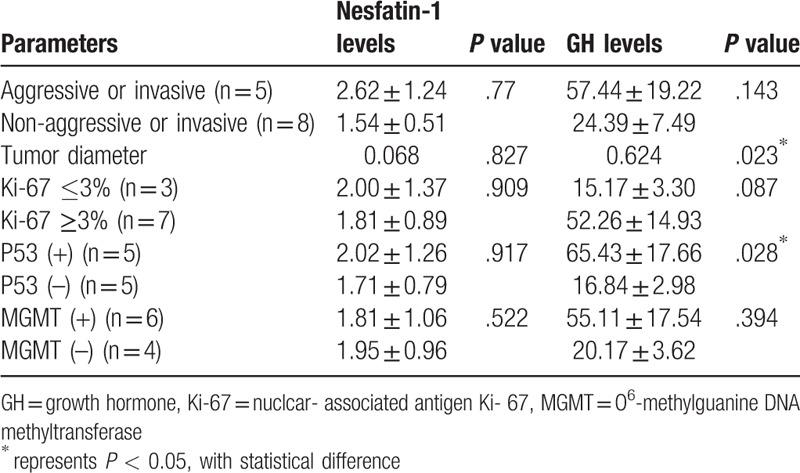
The relationship between serum nesfatin-1 levels and aggressive behaviors in pituitary adenomas.

## 4 Discussion

In this study, our patients with acromegaly suffered from disordered metabolism, and had an elevated levels of nesfatin-1 meanwhile. The increased levels of nesfatin-1 in acromegaly might due to 2 reasons: the tumor itself may secret nesfatin-1; alternatively the disordered metabolism caused the increased levels of this hormone. Moreover, the surgical treatment did not normalize the circulating levels of nesfaitn-1 3rd days postoperatively. The half-life of nesfatin-1 was 9 to 10 minutes.[^17^,^18^] The 3 days was long enough for patients to normalize the levels of nesfatin-1 if the elevated levels of nesfatin-1 was produced by the tumor. As there was no change of nesfatin-1, the reason was due to disordered metabolism which caused the elevated levels of nesfatin-1. Moreover, nesfatin-1 could improve the glucose and lipids metabolism in diabetic mice.[^4^,^5^]

Consequently, the elevated levels of circulating nesfatin-1 were a compensatory mechanism to normalize the disordered metabolism in acromegaly. In addition to the regulation of metabolism, nesfatin-1 also participated in cardiovascular actions and gastric mobility, stress, and reproduction.[^3^,^19^,^20^] Recent reports describe nesfatin-1 as molecules with neuroprotective property that relieve oxidative stress.^[21]^ The excess of GH production in patients with acromegaly could lead to diabetes which obesity and oxidative stress are linked.[^12^,^22^,^23^,^24^]

Compared with the control group, our acromegalic patients showed a significant positive correlation of nesfatin-1 with diastolic pressure. The increased plasma nesfatin-1 levels were observed in hypertension patients, and associated with a higher risk of hypertension.^[25]^ So our results suggested that elevated levels of nesfatin-1 could contribute a higher risk of hypertension in acromegaly.

Recent studies suggested a role of nesfatin-1 in the development of tumor. The intratumoral nesfatin-1 was heavily expressed in colon cancer,^[26]^ prostate cancer,^[27]^ breast cancer,^[28]^ and renal carcinoma.^[29]^ The higher levels of nesfatin-1 was also associated with short overall survival^[27]^ and lymph node metastasis.^[28]^ These results indicated that nesfatin-1 promote cancer progression, and served as a poor prognostic marker. Consequently, increased levels of the circulating nesfatin-1 in acromegaly were likely to promote cancer progression. Although we did not observe the correlations of circulating nesfatin-1 with predictive biomarkers of aggressive pituitary adenomas (Table 2). Contrastly, we observed a significant relationship between serum GH level and tumor's size or p53 mutant expression, corresponding to the previous results.[^30^,^31^]

Still our study placed importance of curing endocrine diseases in acromegaly. The disordered metabolism in acromegaly might promote tumor progression by causing some hormones imbalance.

Our study has, however, several limitations. First, the limited cases in our study might be a shortcoming. Second, we haven’t explored the exact mechanism. Sample size may cause some results to be insignificant or irrelevant and more studies should detect the role of nesfatin-1 in acromegaly.

In conclusion, we first recorded the increased levels of circulating nesfatin-1 in acromegaly. The altered levels of nesfatin-1 were most likely to be caused by the energy imbalance. At last, we suggested that GH excess could promote the progression of pituitary adenomas.

## Author contributions

Conception and design were contributed by CY and NL. Collection and assembly of data were contributed by YY, SH, ZY and PW. Data analysis and interpretation were contributed by YY, SH, ZY and PW. Manuscript writing was contributed by all authors. All authors gave final approval of the manuscript.
